# Hepatocellular carcinoma in an adult patient with congenital absence of the portal vein type II: A case report

**DOI:** 10.1002/jgh3.12312

**Published:** 2020-02-10

**Authors:** Hiroki Bessho, Satoshi Tanaka, Akio Ishihara, Shinya Kato, Reishi Toshiyama, Naoki Hama, Kiyoshi Mori, Masayuki Mano, Atsushi Miyamoto, Hisashi Ishida, Motohiro Hirao, Eiji Mita

**Affiliations:** ^1^ Department of Gastroenterology and Hepatology National Hospital Organization Osaka National Hospital Osaka Japan; ^2^ Department of Surgery National Hospital Organization Osaka National Hospital Osaka Japan; ^3^ Department of Pathology National Hospital Organization Osaka National Hospital Osaka Japan

**Keywords:** congenital abnormalities, congenital absence of the portal vein, hepatocellular carcinoma

## Abstract

Congenital absence of the portal vein (CAPV) is a rare malformation in which intestinal and splenic venous blood bypasses the liver and drains into systemic veins. CAPV is classified into two types based on the absence (type I) or presence (type II) of portal venous flow into the hepatic parenchyma and is associated with multiple other anomalies such as usually benign hepatic tumors. There have been only two case reports describing hepatocellular carcinoma (HCC) in patients with CAPV type II to date. We report the third such patient. A 50‐year‐old woman was referred to our hospital for management of a giant hepatic tumor. Contrast‐enhanced computed tomography (CECT) indicated a huge mass occupying the right lobe of the liver; the radiological diagnosis was HCC. CECT also demonstrated that the superior mesenteric vein (SMV) and the splenic vein (SpV) joined to form a shunt draining into the left renal vein and that a hypoplastic portal vein branched from the confluence of the SMV and SpV and drained into the liver, indicating that the CAPV was type II. Liver resection was successfully performed to treat the HCC, and the pathological diagnosis was well‐differentiated HCC. Seven months after the operation, a recurrent small HCC was detected and treated with radiofrequency ablation without complications. The patient has been carefully followed for 6 months to date without any evidence of further recurrence. Patients with CAPV are predisposed to developing HCC and require close surveillance.

## Graphical abstract

A 50‐year‐old woman was diagnosed with a giant hepatocellular carcinoma (HCC) that was associated with congenital absence of the portal vein (CAPV) type II. Patients with CAPV type II are predisposed to developing HCC and require close surveillance.

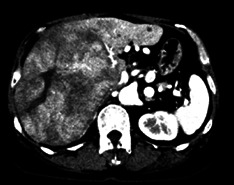

## Introduction

Congenital absence of the portal vein (CAPV), which was first reported by John Abernethy in 1793 and is also known as an “Abernethy malformation,” is a rare anomaly of the splanchnic venous system where intestinal and splenic venous blood bypasses the liver and drains into systemic veins, such as the renal veins, hepatic veins, or inferior vena cava.^1^ CAPV is classified into two types according to the absence (type I) or presence (type II) of portal venous flow into the hepatic parenchyma.^2^ The condition is usually diagnosed in children and is associated with multiple other anomalies such as skeletal and cardiac abnormalities.^3^ Hepatic abnormalities are also reported in patients with CAPV, including biliary atresia, portal hypertension, and a higher incidence of primary hepatic tumors. Hepatic tumors are more frequently discovered in patients with CAPV type I than in those with type II^4^; such tumors mainly comprise focal nodular hyperplasia and other benign types.^5^

Here, we describe a rare CAPV type II in a 50‐year‐old woman with a giant well‐differentiated hepatocellular carcinoma (HCC) that was successfully treated with liver resection. To our knowledge, this is the third reported incidence of CAPV type II that is associated with HCC development.

## Case report

**Figure 1:**
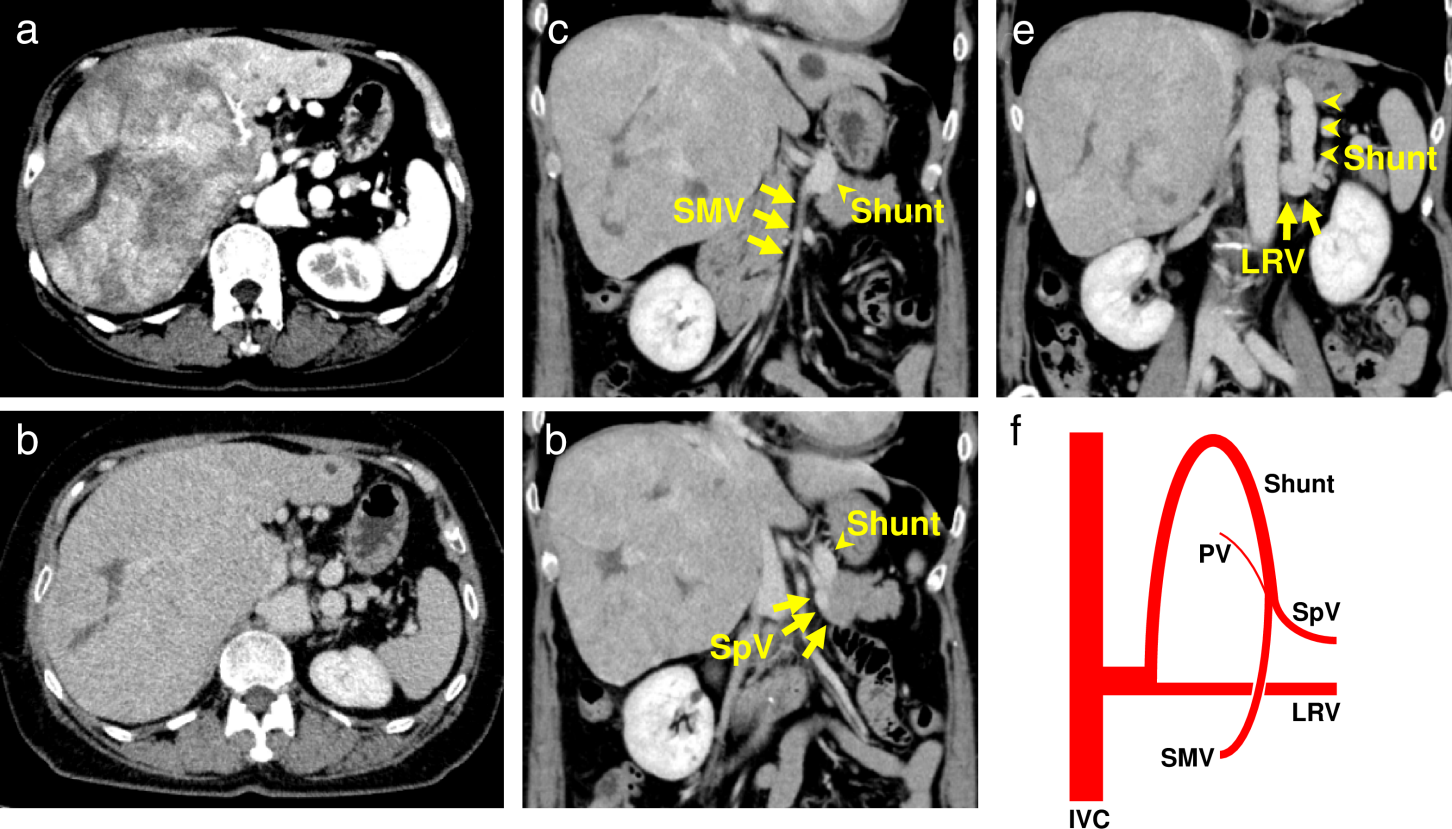
Contrast‐enhanced computed tomography imaging of the abdomen in the arterial phase (a) and the portal venous phase (b–e). The tumor occupying the right robe of the liver showed inhomogeneous hyperenhancement, with a low concentration area in the center in the arterial phase (a) and uniform isoenhancement in the portal venous phase (b). The superior mesenteric vein (SMV) and splenic vein (SpV) joined to form a shunt, draining into left renal vein (LRV) (c–e). Illustration of major abdominal veins (f). IVC, inferior vena cava.

A 50‐year‐old woman was referred to our hospital for management of a giant hepatic tumor. She had been well until 1 month prior, at which time she experienced a sensation of abdominal distension and noticed a loss in appetite. She underwent abdominal ultrasonography at another hospital, whereupon a giant hepatic mass was discovered in the right lobe. The patient was then referred to our hospital for further investigation and treatment of the hepatic tumor. Physical examination demonstrated hepatomegaly; her laboratory test results at the initial visit were as follows: liver function tests showed evidence of nonicteric cholestasis with mild transaminase enzyme level elevation. Screening for viral hepatitis yielded negative results. Levels of the liver‐specific tumor marker “protein induced by vitamin K absence‐II” were highly elevated. No hyperammonemia was detected, and no episodes or signs of encephalopathy were noted. Abdominal ultrasonography showed a giant hypoechoic mass measuring 139 × 120 mm in the right lobe of the liver. Contrast‐enhanced computed tomography (CECT) demonstrated a huge mass that occupied the right lobe of the liver and showed inhomogeneous hyperenhancement in the arterial phase, as well as uniform isoenhancement in the portal venous phase (Fig. 1a,b). A low‐concentration area indicated the presence of necrosis in the tumor center, and a radiological diagnosis of HCC was therefore posited. The superior mesenteric vein (SMV) and the splenic vein (SpV) joined to form an end‐to‐side shunt and drained into the left renal vein (Fig. 1c–e), while a hypoplastic portal vein branched from the confluence of the SMV and SpV and drained into the liver. These findings suggested the presence of CAPV type II (Fig. 1f). Cardiac ultrasonography detected no apparent heart malformations, and cardiac function was normal.

**Figure 2:**
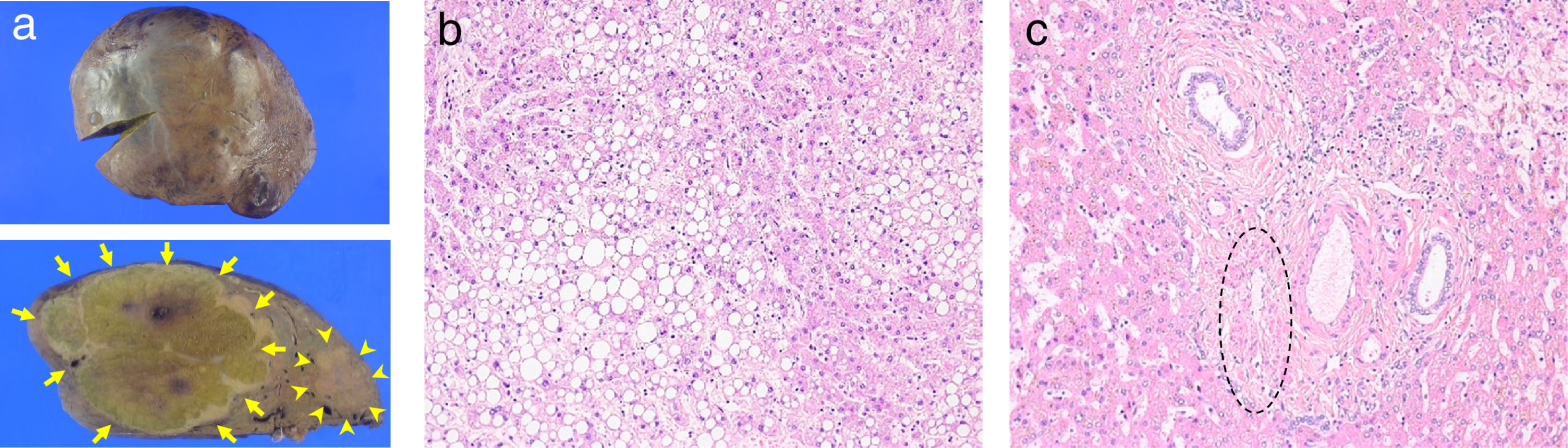
Macro‐ (a) and microscopic (b, c) findings of the resected liver specimen. (a) Macroscopic view of the resected liver specimen (upper) and its cross‐sectional view (lower). Two hepatocellular carcinoma nodules were contained in the specimen (arrows and arrowheads). (b) Hematoxylin and eosin staining of the tumorous lesion (×200). (c) Hematoxylin and eosin staining of the uninvolved liver parenchyma (×200). A hypoplastic portal vein was observed in the portal tract (demarcated by the dashed circle).

A right hepatectomy was performed; during the surgery, dissection of the porta hepatis showed that the hypoplastic portal vein drained into the liver. The SMV and SpV formed a confluence flowing into the left renal vein. The resected liver specimen weighed 1700 g (Fig. 2a).

Microscopic examination indicated that the resected specimen contained two well‐differentiated HCCs (14.5 × 8.0 cm and 3.3 × 2.5 cm) (Fig. 2a). Both were composed of small hepatocytes arranged in trabeculae that were two or three cells thick with minimal nuclear atypia, a slight increase in the nuclear‐to‐cytoplasmic ratio, and lipid droplets (Fig. 2b). The adjacent uninvolved liver parenchyma was noncirrhotic. The portal tracts contained a paired artery and bile duct of approximately equal size, although the portal vein was hypoplastic and reduced in size (Fig. 2c).

The patient had an uneventful postoperative course and was discharged on postoperative day 17. Seven months after the operation, a small recurrent HCC nodule was detected in the remnant liver on follow‐up gadolinium‐ethoxybenzyl‐diethylenetriamine pentaacetic acid (Gd‐EOB‐DTPA)‐enhanced magnetic resonance imaging (MRI); this lesion was successfully treated with radiofrequency ablation. She has been carefully followed for 6 months to date without any evidence of further recurrences.

## Discussion

CAPV is a rare condition in which the intestinal and splenic venous drainage routes bypass the liver and deposit directly into systemic veins through various venous shunts.^1^ During embryological development, the portal vein is normally formed between the 4th and 10th weeks of embryogenesis by selective involution of the peri‐intestinal vitelline venous loop; excessive involution can result in agenesis or hypogenesis of the portal vein.^3^

CAPV is classified into two types based on whether the portal vein is present.^2^ Type I is characterized by the complete absence of the intrahepatic portal vein and thus the lack of hepatic portal venous flow, whereas patients with type II have a partial portal flow into the liver through a hypoplastic portal vein. Type I is further subclassified into Ia (in which the SpV and SMV drain separately into a systemic vein) and type Ib (in which the SpV and SMV merge to form a shunt into a systemic vein). In our patient, abdominal CECT imaging indicated that a hypoplastic portal vein drained into the right lobe of the liver and that the SMV and SpV joined to form an extrahepatic main portal vein that drained into the left renal vein. Intraoperative observation of the liver and microscopic examination of the resected liver specimen also indicated portal vein hypogenesis. These findings demonstrated that our patient had CAPV type II.

CAPV can have various clinical presentations ranging from no symptoms to systemic manifestations such as encephalopathy, hepatopulmonary syndrome, or symptoms related to hepatic masses. CAPV is often associated with metabolic disorders such as hypergalactosemia and hyperammonemia,^3^ as well as other anomalies including skeletal and cardiac abnormalities.^6^ The incidence of CAPV has been estimated to be 1 per 30 000 births.^7^ Patients without symptoms are also sometimes diagnosed based on incidental radiological findings.^8^ In our patient, CAPV was discovered owing to an incidental computed tomography scan performed to diagnose her giant hepatic tumor. She had no symptoms of this disorder or any anomalies.

CAPV often causes hepatic tumors such as focal nodular hyperplasia, nodular regenerative hyperplasia, hemangioma, and HCC. In most cases, hepatic tumors in patients with CAPV are benign.^3^ Kobayashi *et al*. reported that patients with CAPV type I have a significantly higher prevalence of hepatic tumors and other anomalies than do those with type II.^4^ To date, two patients with CAPV type II have been reported to develop HCC. Franchi‐Abella *et al*. reported a 5‐year‐old girl who had multiple liver tumors associated with CAPV type II and had experienced learning disabilities. The liver tumors were not treated, and after she died at the age of 19 years, postmortem examination indicated that the tumors were multifocal HCC and multiple adenomas.^7^ Witters *et al*. reported an HCC within an adenoma in a 42‐year‐old woman with CAPV type II.^9^ Our patient is the third with CAPV type II in whom HCC was discovered.

It remains unclear how CAPV causes HCC. Previous investigators have hypothesized that the absence of portal venous flow compensatively increases the hepatic arterial flow, which might provide a permissive environment for tumor development,^4^ although this remains to be verified. The pathological analysis showed that our patient had no signs of chronic liver disease or liver cirrhosis in the nontumorous liver parenchyma. In their review, Desai *et al*. reported that 20% of HCCs arise in noncirrhotic livers, where their risk factors are non‐alcoholic fatty liver disease, chronic viral hepatitis, inherited diseases such as alpha‐1 antitrypsin deficiency, and intake of genotoxic substances such as aflatoxin B1.^10^ Our patient had no such identifiable risk factors. Further studies are required to elucidate risk factors for HCC development in patients with CAPV.

The lifetime risk for HCC development in patients with CAPV is yet to be determined owing to the rarity of the disease and lack of longitudinal studies. Konstas *et al*. suggested that the risk of HCC among patients with CAPV may be similar to that among alcoholics or patients with hepatitis C‐induced cirrhosis.^8^ Hence, periodic surveillance for HCC may be necessary for patients with CAPV as frequently as it is for those with cirrhosis.

In conclusion, ours is the third reported patient with CAPV type II to develop HCC, for which she underwent a radical resection. A postoperative recurrent lesion in the remnant liver was successfully treated with radiofrequency ablation. Taken together, the data suggested that patients with CAPV type II are predisposed to HCC and therefore require close surveillance.
